# Quality of life of parents raising children with pervasive developmental disorders

**DOI:** 10.1186/1471-244X-12-119

**Published:** 2012-08-20

**Authors:** Atsurou Yamada, Misuzu Kato, Miyoshi Suzuki, Masako Suzuki, Norio Watanabe, Tatsuo Akechi, Toshi A Furukawa

**Affiliations:** 1 Department of Psychiatry and Cognitive-Behavioral Medicine, Nagoya City University Graduate School of Medical Sciences, Mizuho-cho, Nagoya, Mizuho-ku, Japan; 2 Children’s Mental and Physical Development Center, 100 Aza Nakahara, Nakano–Cho, Toyohashi, Aichi, Japan; 3 Iwanishi nursery school, 1-104 Kitahara Takashi-Cho, Toyohashi, Aichi, Japan; 4 Department of Health Promotion and Human Behavior (Cognitive-Behavioral Medicine), Kyoto University Graduate School of Medicine/School of Public Health, Yoshida Konoe-cho, Kyoto, Sakyo-ku, Japan

## Abstract

**Background:**

It has been reported that parents of children with pervasive developmental disorders (PDDs) face higher levels of stress. The aims of the present study were; (i) to evaluate the quality of life (QOL) of parents caring for their children with PDDs, and (ii) to explore the correlates of their QOL.

**Methods:**

A consecutive sample of parents of children with PDDs aged 6 to 15 were approached. The MOS 36-item Short-Form Health Survey (SF-36) was used to measure the QOL of the parents by eight subscales and two summary measures. Parents’ personality and marital relationships were assessed with the NEO Five Factor Inventory and the Intimate Bond Measure, respectively. We characterized the parents’ SF-36 profiles in comparison with the national normative scores and explored variables which correlated with their summary measures.

**Results:**

Participants were 147 mothers and 122 fathers of 158 children with PDDs. Mothers had significantly lower scores in the areas of Role Physical (RP) Social functioning (SF), General health perceptions (GH), Vitality (VT), Role emotional (RE) and Mental Health (MH) than those among the general female population. The maternal mental component summary (MCS) was also significantly lower, but maternal physical component summary (PCS) and paternal PCS and MCS scores were not lower. Maternal PCS and MCS scores were both significantly associated with the high Care and the low Control scores, but regarding fathers only the paternal PCS scores were significantly associated with the low Control scores. Maternal PCS and MCS and paternal MCS scores were significantly associated with the high Agreeableness scores and the low Neuroticism scores. Multiple regressions have shown that Neuroticism was significantly related to the low MCS scores of mothers and fathers. Next, Care was related to maternal high PCS, and Control was related to maternal low MCS and paternal low PCS.

**Conclusions:**

The mothers of children with PDDs had lower QOL scores than those of the Japanese general population especially in mental domains. Impairment of the maternal QOL is significantly associated with the personality tendency of the parents and relationships with their partners.

## Background

As the concept of the autistic spectrum becomes more wide spread, pervasive developmental disorders (PDDs) as a whole are receiving more attention than the narrowly defined autistic disorder *per se.* Because the prevalence of PDDs is 11.3 per 1000 children aged 8 years according to a recent report [1], it is far more prevalent than narrowly defined autistic disorders. As PDDs become more common, the distress of rearing children with PDDs is receiving wider attention.

A number of studies have established that parents of children with autism face higher levels of stress than parents of children with other chronic diseases or intellectual disorders. The stress is higher among mothers than fathers [2-8], and mothers reported more anxiety [9] or more depression [10-13]. Recently the distress of the parents with PDDs including Asperger’s disorder and any PDD not otherwise specified (PDDNOS) are receiving more attention. Many studies show these parents also have high distress [14]. It has been reported that parents of children with PDDs have impairment in their physical activity and social relationships and a worse overall perception of their quality of life (QOL) and health compared with parents of healthy children [15,16]. There is another study which suggested that the parents had poorer scores in all dimensions of health except physical health and had more psychological disorders [17]. Thus the parents of children with PDDs seem to have similar impairment in various domains of their QOL as the parents of children with autism.

The psychological adaptation of parents rearing children with PDDs has been recognized as being partly determined by the symptoms behavior problem [6,9], cognitive impairment [18],self-harm [2], sleep problems [19] and so on of the children, and partly determined by psychosocial factors [20-22]. On the other hand, social support has been identified as one of important moderators ameliorating the stress of parents of children with PDDs [23-27]. It has been reported that social support from the spouse in particular might be a predictor of decreasing complaints related with somatic symptoms and increasing feelings of accomplishment in parenting [28]. It is reported that parent who employed emotion-focused coping strategies had a greater disturbance in their QOL, and that parents to whom social support was less available employed such strategies [29].

In addition to this, personality characteristics such as locus of control and hardiness have also been identified as one of moderators which contribute to the well-being of mothers of children with PDDs [22-24,27,28].

There are many measures to assess the distress of parents. The QOL score is an important measure of individual well-being and it is considered a key variable in evaluating parents’ adaptation to their children’s disabilities [15-17,22,29-32]. For example, it is reported that a mother’s feeling, history of chronic disease and religion were related to the QOL in mothers of children with autism [32]. Another previous study showed the parents of children with autism report a lower QOL and a greater level of child caring burden than the parents of children with attention deficit/hyperactivity disorders (ADHD) [31].

To the present, exactly what characteristics of the parents are related to the impairment of their QOL remains to be elucidated. Identifying these factors could contribute to better care for parents of PDD-affected children. The purpose of this study was to evaluate the QOL of parents caring for their children with PDDs and to explore their correlates.

In this report, we first assessed the QOL of parents caring for children with PDDs in the physical and psychological components of their QOL. Second, we explored the correlates with the impairment of QOL from the point of view of the relationship between mothers and fathers, and their personality characteristics.

## Methods

We used data from our preceding study [14].

### Participants

The details of the purposes and procedures of the study were explained to a consecutive sample of families of children with PDDs who visited the Outpatient Pediatric Neuropsychiatry Clinic of the Toyohashi Municipal Hospital, Japan, during the 3-month period from September to November 2006. The parents often had two or more target children. Because all of their children may affect their parents’ QOL, we tried to obtain the data of all siblings if they were eligible and visiting our clinic.

Parents of children satisfying the following criteria were considered eligible for the study.

1) Children were of elementary school or junior high school age (6 to 15 years old);

2) Children were diagnosed as having autistic disorder, Asperger’s disorder or PDD-NOS according to the DSM-IV diagnostic criteria [33] at our outpatient clinic;

3) Siblings of the children who satisfied 1) and 2) above and were visiting our outpatient clinic were also included even if they themselves did not visit the clinic during the period mentioned above.

We excluded:

1) Children for whom parents did not serve as the main care providers at home;

2) Children or parents whose mother tongue was not Japanese; and

3) Children with tuberous sclerosis.

After giving the necessary explanations at the outpatient clinic, we handed a questionnaire to those consenting to the study, requesting them to mail it back after completing it at home.

The study protocol was approved by the Ethics Committee of Toyohashi Municipal Hospital. Written informed consent was obtained from each of the parents who filled out and returned the self-report style questionnaire.

Data pertaining to the characteristics of individual children, such as age, sex, diagnosis, IQ (intelligence quotient) or DQ (developmental quotient), the characteristics of the autistic disorder and the time of the first visit to the clinic, were collected at the outpatient clinic. The diagnosis of the mental disorder was confirmed by the lead author (a child psychiatrist who had been engaged in child psychiatry for more than 7 years), based on the DSM-IV diagnostic criteria [34]. The IQ was measured with the Wechsler Intelligence Scale for Children-Third Edition (WISC-III) [35,36], and the DQ was measured with the Kyoto Scale of Psychological Development 2001 [37].

### Measures

#### The MOS 36-item Short-Form Health Survey (SF-36)

SF-36 is a self-report questionnaire to assess general QOL. It contains 36 items that constitute 8 measures of Physical Functioning (PF), Role Physical (RP), Bodily Pain (BP), Social Functioning (SF), General Health Perceptions (GH), Vitality (VT), Role Emotional (RE), and Mental Health (MH). It also provides two summary measures, the Physical Component Summary (PCS) and the Mental Component Summary (MCS). The PCS refers to general factors of physical health, and it is associated with PF, RP, BP, GH and VT. The MCS refers to psychological health, and it is associated with MH, RE, SF, VT and GH. The total result is shown in each of the 8 measures by a standard score which ranges from 0 to 100, and the higher the score, the higher the QOL. The average and standard deviation of PCS and MCS scores of the general Japanese population are 50 and 10 respectively. The Japanese version has shown good reliability and validity in the general population of Japan [38,39].

#### Intimate Bond Measure (IBM)

This measure is designed to evaluate the nature of the subject’s relationship with their marital partner. It is a self-report measure for evaluation of two aspects of marital relationships (Care and Control), and is composed of 24 questions. The Care dimension reflects care expressed emotionally as well as physically, with constructs of warmth, consideration, affection and companionships. The Control dimension suggests domination, instructiveness, criticism, authoritarian attitudes and behaviors. The respondent selects one of the four answers (3 = yes, 2 = somewhat yes, 1 = relatively no, 0 = absolutely no). Each aspect consists of 12 questions, therefore, the total score ranges from 0 to 36, and a high score means a tendency to Care or Control, respectively. The questions pertain to the recent situation, without specifying the period covered. The reliability and validity of this measure have been confirmed [40,41].

#### NEO Five Factor Inventory (NEO-FFI)

The NEO-FFI is a concise measure of the five domains of personality according to the five-factor model. Twelve items are provided for each of the five dimensions of Neuroticism, Extraversion, Openness, Agreeableness, and Conscientiousness. Each item is answered on a five-point Likert scale (0 = strongly disagree, 1 = disagree, 2 = neutral, 3 = agree, 4 = strongly agree), so that each dimension is given a score from 0 to 48 and a high score means one has a higher tendency towards each personality. The Japanese version has shown good reliability and validity in the general population of Japan [42].

### Brief demographic self-report questionnaire and family profiles

The questionnaire included information about the age, occupation, education, and history of psychiatric treatment of the parents and the presence/absence of additional children with PDDs in the family.

### Analyses

First, we calculated the mean values of the 8 subscales of SF-36 and compared them with those of age- and sex-matched general population subjects sampled in Japan by way of an unpaired *t*-test. The comparison group was sampled from 300 areas in Japan, stratified by its size of population, from each of which 15 people aged between 20 to 80 of age were randomly sampled. Finally the comparison group consisted of 1572 women and 1394 men [43]. We extracted the age- and sex-matched data from them. We also compared them between mothers and fathers by paired *t*-test. To evaluate the influence of number of rearing children with PDDs on PCS and MCS, we compared those scores of parents rearing one child with PDD and two or more children with PDDs. Next we explored variables which correlated with the PCS and MCS scores using Pearson’s correlation coefficients. The independent variables we explored were the personality traits as measured by NEO-FFI and the husband-wife relationship as measured by IBM. Finally, a multiple regression analysis of the PCS and MCS scores of the mother and father was conducted by the forced entry method. We used SPSS for Windows 17.0 [44] for all data analyses. *p* < .05 was regarded as denoting statistical significance in all analyses.

## Results

### Subjects

We explained the study and gave questionnaires to a consecutive sample of 198 families. Responses were collected from 147 families (74.2%) including 147 mothers and 122 fathers, covering 158 children. Table 1 shows the demographic data of the parents (Table 1). The 158 children consisted of 42 with autistic disorders (35 boys), 35 with Asperger’s disorders (29 boys) and 81 with PDD-NOS (65 boys). One hundred twenty-four families including 124 mothers and 102 fathers had only one child diagnosed with PDD. The children ranged in age from 6 to 15 years and the mean age was 9 years 1 month (SD = 2 years 6 months). The distribution of the IQ/DQ was as follows: Ninety children were over 70, 26 children were between 50 and 69, 18 children were between 35 and 49, 7 children were between 20 and 34, and 6 children were less than 20.

**Table 1:** Demographic data of parents and children

	**Mother**	**Father**
Number	147	122
Age(years)	38.3 (SD = 4.6)	41.0 (SD = 5.7)
A parent–child relationship		
Real	147	121
Foster	0	1
Marriage		
Married	138	122
Divorced	9	0
Work		
Housework or out of work	74	1
Under 40 hours per week	64	5
Over 40 hours per week	9	116
Education		
Up to junior high school	3	6
High school	77	66
Junior college or beyond	67	50
Time spend with children		
Almost never	0	5
Less than one hour everyday	1	17
Less than one hour on weekdays but more on weekends	2	57
More than one hour everyday	25	26
More than a few hours everyday	119	17
Number of children with PDDs		
One	124	102
Two or more	23	20
History of psychiatric treatment		
Positive	27	6
Negative	120	116

Abbreviations: PDD: pervasive developmental disorder; SD, standard deviation.

### QOL of parents

Table 2 shows the scores of SF-36 (Table 2). The comparison groups consisted of 255 women aged from 30 to 39, and 241 men aged from40 to 49 who represented random samples from all areas in Japan mentioned above [43]. The mean scores of mothers were significantly lower than those of the general female population in six domains (*p* < .01) especially in psychological areas. On the other hand, in the fathers, the mean scores were significantly lower only in 1 domain (*p* < .05). The scores were lower among mothers than among fathers in 6 domains (all *p* < .01) especially in psychological areas.

**Table 2:** The The MOS 36-item Short-Form Health Survey scores of parents and comparison with those of general population

	**Mothers**			**Fathers**			**Paired sample**	
	**n**	**Average (SD)**	**Difference in average of general female population and mothers (95%CI)**	**n**	**Average (SD)**	**Difference in average of general population and fathers (95%CI)**	**n**	**Difference in average of mothers and fathers (95%CI)**
Physical Functioning	147	90.8 (10.7)	−0.8 (−3.1~1.5)	122	92.9 (10.9)	0.8 (−1.5~3.1)	122	−1.7 (−4.3 ~ 0.9)
Role Physical	146	76.2 (37.4)	−11.8** (−17.4~ − 6.2)	122	88.9 (25.5)	−3.6 (−7.8~0.6)	121	−12.0** ( − 19.1 ~ −4.9)
Bodily Pain	147	68.7 (23.9)	−3.8 (−8.6~1.0)	122	80.4 (20.7)	3.7 (−0.9~8.3)	122	−11.7** (−17.6 ~ −5.8)
General Health Perceptions	147	59.6 (20.1)	−5.3** (−9.1~−1.5)	122	61.5 (17.5)	−2.5 (−6.2~1.2)	122	−1.6 (−6.8~3.6)
Vitality	147	49.4 (23.5)	−9.3** (−13.6~ − 5.0)	122	56.8 (17.6)	−4.3* (−8.5~ − 0.1)	122	−6.9** (−11.9~ − 2.0)
Social Functioning	147	76.7 (24.5)	−8.1** (−12.5~ − 3.7)	122	89.1 (16.2)	0.7 (−3.1~4.5)	122	−11.6** (−16.6~ − 6.6)
Role Emotional	146	69.9 (39.0)	−15.3** (−21.1~ − 9.5)	122	88.3 (25.0)	−2.0 (−6.3~2.3)	121	−17.4** (−24.8~ − 9.9)
Mental Health	147	58.5 (22.5)	−11.1** (−15.3~ − 6.9)	122	69.1 (16.9)	−0.8 (−4.8~3.2)	122	−9.5** (−14.3~ − 4.8)
Physical Component Summary	146	50.3 (6.9)	−1.0 (−2.5~0.5)	122	51.1 (5.5)	−1.2 (−2.6~0.2)	121	−0.9 (−2.4~0.6)
Mental Component Summary	146	42.9 (11.9)	−5.0** (−7.2~−2.8)	122	49.4 (8.0)	0.3 (−1.7~2.3)	121	−6.1** (−8.5~ − 3.7)

Abbreviations: CI, confidence interval; SD, Standard Deviation.

*p < .05; ** *p* < .01.

The PCS and MCS average scores of 123 mothers with one child with PDD were 50.6 (Standard Deviation [SD] = 6.7) and 43.3 (SD = 11.8), and those of 23 mothers with two or more children with PDDs were 48.7 (SD = 8.0) and 41.0 (SD = 12.7) respectively. There were no significant differences between the mothers with one child with PDD and two or more children with PDDs. The PCS and MCS average scores of 102 fathers with one child with PDD were 50.9 (SD = 5.6) and 49.4 (SD = 7.9), and those of 20 fathers with two or more children with PDDs were 52.3 (SD = 5.4) and 49.2 (SD = 8.5) respectively. There were no significant differences, either.

### Correlates of QOL

Table 3 shows the correlations between the PCS and MCS scores of SF-36 and the husband-wife relationship as measured with the IBM scores, the personality traits as measured with the NEO-FFI and other factors (Table 3). Maternal PCS and MCS scores were both significantly associated with the high Care scores and the low Control scores, but in the fathers only the PCS scores were associated with the low Control scores. Maternal PCS and MCS and paternal MCS scores were associated with the high Agreeableness scores and the low Neuroticism scores. In case of mothers, both the attitudes from their spouses and personal traits were significantly associated with physical and psychological QOL. In case of fathers, the attitudes from their spouses were associated with only physical QOL and personal traits were significantly associated with only psychological QOL.

**Table 3:** Correlations between Maternal PCS and MCS scores and Intimate Bond Measure scores or NEO Five Factor Inventory scores

	**Mothers**			**Fathers**		
	**n**	**PCS**	**MCS**	**n**	**PCS**	**MCS**
Intimate Bond Measure scores ^a^						
Care by spouse	137	.293**	.178*	122	.117	−.018
Control by spouse	137	−.173*	−.187*	122	−.278**	−.173
NEO Five Factor Inventory scores of mothers and fathers ^b^						
Neuroticism	146	−.165*	−.468**	122	−.150	−.604**
Extraversion	146	.209*	.394**	122	−.041	.176
Openness	146	−.152	−.026	122	.098	.102
Agreeableness	146	.170*	.268**	122	.002	.317**
Conscientiousness	146	.084	.030	122	.035	.259**
Time spend with children						
Mothers	146	.012	−.041	122	.029	−.085
Fathers	121	.133	.119	122	.065	.216*
Work time						
Mothers	146	.234**	.113	122	−.085	−.010
Fathers	121	−.136	−.111	122	.258**	−.077
Education	146	−.058	−.183*	122	.101	.007
Living together						
Paternal grandfather	146	.196*	.029	122	.074	−.131
Paternal grandmother	146	.184*	.092	122	−.009	−.094
Maternal grandfather	146	−.008	−.025	122	.029	−.085
Maternal grandmother	146	−.002	.036	122	−.027	.016

Abbreviations: PCS, Physical Component Summary; PDD, pervasive developmental disorder; MCS, Mental Component Summary.

^a^ Each Item is a four-point Likert scale (3 = yes, 2 = somewhat yes, 1 = relatively no, 0 = absolutely no). The total score ranges from 0 to 36.

^b^ Each Item is a five-point Likert scale (0 = strongly disagree, 1 = disagree, 2 = neutral, 3 = agree, 4 = strongly agree). The total score ranges from 0 to 48.

* p < .05; ** *p* < .01.

In addition to this, the work time of mothers and fathers were significantly related to the high PCS scores of them respectively. Living with paternal grandparents was significantly related to the maternal high PCS scores. Parents working for more time tended to show higher physical QOL. The help from paternal grandparents may heighten the mothers’ physical QOL. On the other hand, maternal education was associated with the high MCS scores. Mothers having an educational background may have resilience in the psychological domains.

Multiple regressions have shown that Neuroticism was significantly associated with the low psychological component scores of mothers and fathers. Next, Care was significantly associated with the high physical component scores of mothers, and Control was significantly associated with the low psychological component scores of mothers and the low physical component scores of fathers (Table 4).

**Table 4:** The correlations between Intimate Bond Measure scores, NEO Five Factor Inventory scores and Parental PCS and MCS scores

	**Standardized Coefficients (β)**	***T***	**Adjusted R**^**2**^
Maternal PCS scores		6.684**	.141
Care	.238	2.734**	
Control	−.092	−1.059	
Neuroticism	−.011	−.116	
Extraversion	.086	.862	
Agreeableness	.067	.735	
Work time of mothers	.156	1.825	
Living with paternal grandfather	.177	1.217	
Living with paternal grandmother	.005	.031	
Maternal MCS scores		4.495**	.273
Care	.017	.215	
Control	−.174	−2.195*	
Neuroticism	−.321	−3.646**	
Extraversion	.219	2.512*	
Agreeableness	.022	.258	
Education of mothers	.117	1.539	
Paternal PCS scores		7.319**	.123
Control	−.266	−3.121**	
Work time of fathers	.245	2.878**	
Paternal MCS scores		8.802**	.402
Neuroticism	−.549	−6.776**	
Agreeableness	.088	1.143	
Conscientiousness	.071	.953	
Time fathers spend with children	.205	2.884**	

Abbreviations: PCS, Physical Component Summary; MCS, Mental Component Summary.

* *p* < .05; ** *p* < .01.

The personal traits were important predictor of parental psychological QOL. On the other hand, the spouse’s attitude was important predictor of paternal physical QOL and maternal psychological QOL.

## Discussion

This is the first study that has thrown light on the relationships between the QOL of parents rearing children with PDDs and their psychosocial characteristics.

First, this study has revealed that mothers of children with PDDs had low QOL scores in several mental domains. Almost all previous studies have shown impairment of QOL among such parents. There is a report that parents of children with autism spectrum disorders had significant poorer self-perceived health except for physical health dimensions [17], and our obtained results are consistent with the findings regarding mothers. According to the previous studies, the children’s disabilities place a higher burden on the mothers among the parents of children with high functioning autism or Asperger’s disease [45,46], and there is a tendency for the mothers to suffer stronger impairment than the fathers in terms of the QOL [15,16]. Our results are largely in line with these previous reports.

The mothers of children with PDDs seem to have lower QOL than those of children with other chronic diseases because of their children’s symptoms and behavior problems. PDDs seem to place greater burden on the parents in comparison with other developmental disorders. For example, the mothers of children with PDDs tend to have a lower QOL in the psychological domain than the mothers of children with mental retardation [15]. Parents of children with autism report a greater level of child caring burden, less frequent attendance of religious service, more school days missed, more repeated grades, less participation in activities/events, and less involvement in community services compared to parents of children with ADHD [31].

In this study, maternal QOL were significantly related to high Care scores and related to low Control scores in both physical and psychological areas. It has been generally indicated that social support is one of many important factors which contribute to the positive adaption of parents [21,23-25,28]. In particular, support from their partners seems to be associated with positive maternal outcomes [11,27,47]. The quality of the marriage has been reported to increase the ability to cope with the stress of parenting an autistic child [22], so that social support from their spouses contributes to positive maternal adaptation to a great extent. For example, single mothers appear to suffer from depression more easily than married mothers [12]. Some reports have suggested that the distress of mothers of PDD-affected children was correlated with perceived control by their spouses [14], expressed support from their spouses was the best predictor of quality of parenting [11], and mothers of autistic children expressed the need for additional support from their spouses [2]. Mothers should receive not only help but also supportive attitude, and should not be under psychological control from their husbands. According to one study, fathers were also much more likely to see themselves as being a reserve source of support for their wives [45].

Moreover, in this study, living with grandparents gave positive maternal QOL scores in physical domains. It is reported that greater family support was associated with increased optimism, and that in turn optimism was associated decreased depression and parenting stress [48]. Living with grandparents might help mothers and play an important role in maternal physical QOL.

Next we found that impairment of the mental QOL was strongly associated with the personality tendencies of the parents. There are rare reports about the association between QOL and personal traits, but a study exists which has reported that the QOL of patients with mood or anxiety disorders is not only determined by the disease or the current health but is also shaped by personality traits [49]. Our results showed that parents with higher Neuroticism scores seem to suffer from a poorer QOL especially in psychological areas. The pattern of personality traits appears to dictate the patterns of the QOL. Knowing the parents’ personality traits may therefore help us decide which interventions can be most successful among the parents.

There are few studies which have reported about the QOL of fathers. It is reported that maternal physical health was poorer than paternal physical health, but that self-related mental health did not differ between mothers and fathers of children with Asperger’s disease or high-functioning autism [16]. It is also reported that fathers of children with PDDs also showed statistically significant lower scores in the social relationship domain compared to fathers of normal developing children and in the psychological domain compared to fathers of children with cerebral palsy, but that mothers showed low scores in more domains than fathers [15]. Thus fathers seem to show less impairment in their QOL than mothers, but the causes are unknown. In this study, the paternal QOL scores were higher than mothers’ QOL, but psychological domains were significantly associated with the attitude by spouse than those of the mothers.

There are some limitations in this study. First, this is a cross-sectional study, precluding any conclusions from being made with regard to causality. Second, we did not control for the parents’ socioeconomic or marital status and number of rearing children when we compared our subjects with the general population, because such information was not available for the normative dataset of SF-36. In an analysis of the QOL, these issues are regarded as limitations because they have been suggested as factors associated with the cores of SF-36 in preceding reports [15]. Third, there might be a sampling bias because all the subjects were parents whose children came to the hospital. One population study reported that mothers of children with autistic disorders showed remarkable strengths in the parent–child relationship and coping with parenting tasks regardless of high stress and poor mental health [20]. Future research should be performed on the general population basis.

Taken together, the results of the present study suggest that we may need to support parents, especially mothers, of children with PDDs in all the aspects of their lives that have been shown to correlate with their QOL. Social support strategies including the spousal support have been identified as the important factor for these mothers. We would need further studies with a similar design to replicate this finding not only in the treatment-seeking subjects but also in the community-dwelling subjects and their families.

## Conclusions

Not only the mothers of children with autistic disorder but also mothers of children with other PDDs had lower QOL scores than fathers, especially in the mental domains. Impairment of maternal QOL is significantly associated with the personality tendency of the mothers and relationships with their partners. Clinicians can deliver better service by paying appropriate attention to the QOL of parents whose children have autistic disorders or other PDDs.

## Competing interests

The authors declare that they have no competing interests.

## Authors’ contributions

All authors have contributed essential parts to the manuscript and are entirely responsible for its scientific content. AY and TAF planned the study. AY, MiS and MK carried out the investigation. AY and TAF analyzed the all data. MaS, NW, TA and TAF critically commented on the draft manuscript. All authors have read and approved the final version of the manuscript.

## Pre-publication history

The pre-publication history for this paper can be accessed here:

http://www.biomedcentral.com/1471-244X/12/119/prepub
